# Metabolic Syndrome, Component Burden, and Incident Gastric Cancer Risk: A Prospective Cohort Study in the UK Biobank

**DOI:** 10.1158/2767-9764.CRC-26-0373

**Published:** 2026-08-10

**Authors:** Gang-Qiang Wang, Shi-Rui Li, Si-Yu Li, Jin-Qiu Yuan, Chang-Hua Zhang

**Affiliations:** 1 Center for Digestive Disease, The Seventh Affiliated Hospital, Sun Yat-sen University, Shenzhen, China.; 2 Department of Health Management, The Seventh Affiliated Hospital, Sun Yat-sen University, Shenzhen, China.; 3 Chinese Health Risk Management Collaboration (CHRIMAC), Shenzhen, China.; 4 Guangdong Provincial Key Laboratory of Digestive Cancer Research, Center for Digestive Disease, The Seventh Affiliated Hospital, Sun Yat-sen University, Shenzhen, China.; 5 Guangdong Provincial Key Laboratory of Digestive Cancer Research, The Seventh Affiliated Hospital, Sun Yat-sen University, Shenzhen, China.

## Abstract

As *Helicobacter pylori* prevalence declines, understanding the contribution of modifiable systemic factors to gastric cancer risk becomes increasingly important. We evaluated the association of metabolic syndrome (MetS), its individual components, and anatomic subsites with incident gastric cancer in 471,540 UK Biobank participants. Multivariable Cox proportional hazards models were used to estimate the risk of overall, cardia, and non-cardia gastric cancers, and restricted cubic splines were used for exploratory nonlinear analyses. Over a mean follow-up of 6.5 years, 332 incident gastric cancer cases were identified. In the fully adjusted model, baseline MetS was associated with increased gastric cancer risk [HR = 1.36; 95% confidence interval (CI), 1.08–1.71]. A positive trend was observed with the accumulation of metabolic components (*P* for trend = 0.033). Subsite analyses showed a directionally positive association for cardia gastric cancer (HR = 1.48; 95% CI, 1.03–2.11) and a nonsignificant positive association for non-cardia gastric cancer (HR = 1.28; 95% CI, 0.95–1.73); however, formal testing found no statistical evidence of subsite heterogeneity (*P* for heterogeneity = 0.547). Exploratory spline analysis suggested a possible nonlinear association for systolic blood pressure. Overall, MetS and the accumulation of its components were associated with a moderately increased risk of gastric cancer. Waist circumference showed the most consistent component-level signal, and further studies are needed to clarify the role of metabolic health in gastric cancer risk assessment.

**Significance:**

In this prospective analysis of 471,540 UK Biobank participants, MetS was associated with a moderately increased risk of incident gastric cancer. A positive trend was observed with cumulative metabolic component burden, and waist circumference showed the most consistent component-level signal. Subsite analyses suggested a stronger point estimate for cardia gastric cancer, but formal testing found no statistical evidence of heterogeneity between cardia and non-cardia tumors.

## Introduction

Although age-standardized gastric cancer (gastric cancer) incidence and mortality rates have declined in many regions (1), partly due to declining *Helicobacter pylori* prevalence, improved food preservation, and changes in traditional lifestyle-related risk factors, gastric cancer remains a major cancer burden (2, 3). Furthermore, changing epidemiologic patterns, such as the increasing or stabilizing rates of cardia gastric cancer and young-onset cases in certain populations (4–7), have heightened clinical interest in identifying noninfectious, modifiable risk factors. As the etiologic landscape of gastric cancer potentially shifts alongside declining *H. pylori* prevalence and changing traditional risk factors (3), the contribution of systemic metabolic abnormalities warrants comprehensive evaluation.

In parallel with these changing patterns, the global prevalence of metabolic syndrome (MetS)—a complex cluster of central obesity, hypertension, dyslipidemia, and dysglycemia—has increased substantially over the past two decades (8). A recent systematic review and modeling analysis estimated that global MetS prevalence increased from 11.9% in 2000 to 28.4% in 2023, affecting approximately 1.54 billion adults worldwide (9). This growing burden makes MetS increasingly relevant to cancer prevention research (10–12). Biologically, MetS may be associated with gastric carcinogenesis through several intersecting pathways, including chronic low-grade systemic inflammation, insulin resistance, altered adipokine secretion, and the mechanical exacerbation of gastroesophageal reflux disease driven by central adiposity (13–17).

Despite emerging evidence linking metabolic dysfunction to gastrointestinal cancers (13, 18), the epidemiologic relationship between MetS and gastric cancer remains incompletely understood. Some previous studies have treated gastric cancer as a single entity, and subsite-specific evidence remains limited and heterogeneous (10, 19–21). Additionally, prior research has often relied on binary cutoffs for individual metabolic components (13, 14, 19), which may obscure complex, nonlinear dose–response relationships. Finally, the potential confounding role of *H. pylori* has frequently been limited by incomplete data in large-scale population cohorts (12, 14, 19–21).

To address these knowledge gaps, we leveraged the large-scale, prospective UK Biobank cohort to investigate the association between MetS and incident gastric cancer. Our primary objectives were to (i) evaluate the overall association between MetS and gastric cancer risk, as well as the impact of cumulative component burden; (ii) explore potential differences in this association across cardia and non-cardia subsites; and (iii) assess the associations of individual continuous metabolic components with gastric cancer risk, using exploratory restricted cubic splines (RCS) to examine potential nonlinear dose–response patterns.

## Materials and Methods

### Study design and participants

The UK Biobank is a large-scale, prospective population-based cohort comprising more than 0.5 million participants of 37 to 73 years of age recruited between 2006 and 2010. The study collected extensive phenotypic data, including sociodemographic assessments, anthropometrics, self-reported lifestyle factors, and clinical diagnoses. Detailed rationales and methodologies of the UK Biobank have been described previously (22). For the current analysis, we sequentially applied the following exclusion criteria: (i) participants with a prior diagnosis of any cancer at baseline (*n* = 26,868), (ii) participants with undefinable MetS status due to missing data on MetS components (*n* = 3,981), and (iii) female participants who were pregnant at the time of baseline assessment (*n* = 138). After these exclusions, a total of 471,540 eligible participants were included in the final analytic cohort (Supplementary Fig. S1).

The UK Biobank study received ethical approval from the North West Multi-centre Research Ethics Committee. All participants provided written informed consent. The present study was conducted in accordance with recognized ethical guidelines.

### Data collection and measurement

Baseline information was obtained through self-administered touch-screen questionnaires, physical measurements, and biological samples. Sociodemographic characteristics included age, sex, ethnicity, educational attainment, and the index of multiple deprivation (IMD) as a proxy for socioeconomic status. Lifestyle factors were comprehensively assessed, including smoking status, alcohol consumption frequency, and dietary habits (evaluated via a validated food frequency questionnaire). Physical activity was quantified using the adapted short International Physical Activity Questionnaire (23).

Physical measurements were performed by trained staff following standardized protocols, including height, weight, waist and hip circumference, and automated blood pressure readings. Laboratory parameters, including serum triglycerides, high-density lipoprotein cholesterol (HDL-C), and fasting blood glucose, were analyzed using a Beckman Coulter AU5800 clinical chemistry analyzer. Medical history and medication use, including antihypertensives, lipid-lowering drugs, and nonsteroidal anti-inflammatory drugs (NSAID), were also recorded to accurately define MetS components.

### Definition of MetS

In our primary analysis, we defined MetS based on the globally unified criteria established by the 2009 Joint Interim Statement (JIS; ref. 24). A participant was classified as having MetS if they met at least three of the following five criteria:

a) Central obesity, defined by waist circumference (≥102 cm for males and ≥88 cm for females).
b) Elevated blood pressure (systolic ≥130 mm Hg and/or diastolic ≥85 mm Hg or current use of antihypertensive medication).
c) Elevated triglycerides (≥1.7 mmol/L or current use of lipid-lowering medication).
d) Reduced HDL-C (<1.03 mmol/L for males and <1.29 mmol/L for females or current use of lipid-lowering medication).
e) Elevated fasting glucose (≥5.6 mmol/L, a previous diagnosis of diabetes, or current use of glucose-lowering medication).

### Assessment of outcomes

The follow-up period spanned from the recruitment date to the date of gastric cancer diagnosis, death, or the study endpoint (October 30, 2015), whichever occurred first. All incident gastric cancer cases were identified through record linkage to national cancer and death registries. Gastric cancer cases were classified as cardia or non-cardia based on International Classification of Diseases-10 codes C16.0 and C16.1–C16.6; no cases with an unspecified subsite were included in the primary subsite analysis.

### Statistical analysis

Baseline characteristics were summarized as means with standard deviations (SD) or frequencies with percentages. Cox proportional hazards models were used to estimate hazard ratios (HR) and 95% confidence intervals (CI) for the association between MetS and incident gastric cancer. Model 1 adjusted for age and sex. Model 2 further adjusted for ethnicity, education, socioeconomic deprivation, smoking status, alcohol consumption, and NSAID use. Model 3 additionally adjusted for physical activity, fruit and vegetable intake, and salt intake. Proportional hazards assumptions were assessed using Schoenfeld residuals.

To evaluate cumulative metabolic burden, the number of MetS components was categorized as 0 to 2, 3, and 4 to 5 components. *P* for trend was estimated by modeling these categories as an ordinal variable. To examine whether associations differed by anatomic subsite, we fitted cause-specific Cox models for cardia and non-cardia gastric cancers, treating the alternative subsite as censored. *P* for heterogeneity was calculated by comparing subsite-specific log HRs using a Wald-type test.

Component-specific associations were evaluated using two approaches. First, each MetS component was modeled as a dichotomous variable based on clinical cutoffs. Second, continuous metabolic biomarkers were standardized to *z*-scores, and HRs were estimated per 1-SD increment. Exploratory RCS models with three knots placed at prespecified percentiles were used to examine potential nonlinear associations between continuous metabolic components and gastric cancer risk (25). Given the limited number of incident gastric cancer cases, these analyses were interpreted cautiously.

Subgroup analyses were conducted by age group, sex, smoking status, and alcohol consumption. Effect modification was assessed by adding cross-product interaction terms between MetS and subgroup variables and comparing models using likelihood ratio tests.

Sensitivity analyses included excluding gastric cancer cases diagnosed within the first 2 years of follow-up, additionally adjusting for family history of cancer, additionally adjusting for preexisting benign gastric diseases, and repeating the analysis in the subcohort with available *H. pylori* serology. Because only eight incident gastric cancer cases occurred in the *H. pylori* subcohort, this analysis was considered exploratory. We also repeated the main analyses using the International Diabetes Federation (IDF) 2005 definition of MetS, which requires central obesity, to assess robustness under an alternative MetS definition.

Missing values in categorical covariates were retained as missing/unknown categories to preserve sample size and avoid list-wise deletion. Complete case analyses were used for models involving continuous metabolic biomarkers.

This was an observational prospective cohort study reported in accordance with the STROBE guidelines; therefore, participants were not randomized to exposure groups, and blinding was not applicable. Baseline demographic characteristics, including age and sex, are summarized in Table 1. No formal *a priori* sample-size calculation was performed because the analysis included all eligible UK Biobank participants who met the prespecified inclusion and exclusion criteria. All analyses were performed using R software version 4.5.2 (R Project for Statistical Computing; RRID: SCR_001905), and two-sided *P* values < 0.05 were considered statistically significant.

**Table 1.** Baseline characteristics according to MetS category.

Characteristic	Overall *N* = 471,540	MetS status	
		No *N* = 298,900	Yes *N* = 172,640
Age (years), mean (SD)	56.3 (8.1)	55.1 (8.1)	58.5 (7.6)
Average follow-up years, mean (SD)	6.5 (1.2)	6.6 (1.2)	6.5 (1.3)
BMI (kg/m^2^), mean (SD)	27.4 (4.8)	25.8 (3.9)	30.2 (5)
Waist circumference (cm), mean (SD)	90.3 (13.5)	85.4 (11.4)	98.9 (12.5)
Systolic blood pressure (mm Hg), mean (SD)	139.7 (19.7)	136.7 (19.6)	144.8 (18.6)
Diastolic blood pressure (mm Hg), mean (SD)	82.3 (10.7)	81 (10.6)	84.5 (10.5)
HDL (mmol/L), mean (SD)	1.4 (0.4)	1.6 (0.4)	1.3 (0.3)
Triglycerides (mmol/L), mean (SD)	1.7 (1)	1.4 (0.7)	2.3 (1.2)
Fasting glucose (mmol/L), mean (SD)	5.1 (1.2)	4.9 (0.6)	5.5 (1.8)
IMD, mean (SD)	17.7 (14)	16.7 (13.4)	19.3 (14.9)
Gender, *n* (%)	​	​	​
Female	254,145 (53.9%)	173,712 (58.1%)	80,433 (46.6%)
Male	217,395 (46.1%)	125,188 (41.9%)	92,207 (53.4%)
Ethnicity, *n* (%)	​	​	​
White	443,398 (94%)	282,075 (94.4%)	161,323 (93.4%)
Non-White or unknown	28,142 (6%)	16,825 (5.6%)	11,317 (6.6%)
Education level, *n* (%)	​	​	​
No college degree	232,935 (49.4%)	147,210 (49.3%)	85,725 (49.7%)
College/university degree	153,039 (32.5%)	108,302 (36.2%)	44,737 (25.9%)
Missing	85,566 (18.1%)	43,388 (14.5%)	42,178 (24.4%)
Smoking status, *n* (%)	​	​	​
Never/unknown	260,223 (55.2%)	174,564 (58.4%)	85,659 (49.6%)
Previous	161,306 (34.2%)	94,092 (31.5%)	67,214 (38.9%)
Current	50,011 (10.6%)	30,244 (10.1%)	19,767 (11.4%)
Alcohol consumption, *n* (%)	​	​	​
Low/none or unknown	145,276 (30.8%)	82,276 (27.5%)	63,000 (36.5%)
Weekly	121,555 (25.8%)	78,207 (26.2%)	43,348 (25.1%)
Frequent	204,709 (43.4%)	138,417 (46.3%)	66,292 (38.4%)
Physical activity (MET), *n* (%)	​	​	​
Moderate/high	309,168 (65.6%)	205,019 (68.6%)	104,149 (60.3%)
Low	71,717 (15.2%)	40,028 (13.4%)	31,689 (18.4%)
Missing	90,655 (19.2%)	53,853 (18%)	36,802 (21.3%)
Fruit/vegetable intake, *n* (%)	​	​	​
≥5 portions/day	177,720 (37.7%)	114,421 (38.3%)	63,299 (36.7%)
<5 portions/day or unknown	293,820 (62.3%)	184,479 (61.7%)	109,341 (63.3%)
Salt intake, *n* (%)	​	​	​
Never/rarely or unknown	261,900 (55.5%)	168,373 (56.3%)	93,527 (54.2%)
Sometimes	132,088 (28%)	83,320 (27.9%)	48,768 (28.2%)
Usually/always	77,552 (16.4%)	47,207 (15.8%)	30,345 (17.6%)
NSAID use, *n* (%)	​	​	​
No or unknown	283,768 (60.2%)	199,284 (66.7%)	84,484 (48.9%)
Yes	187,772 (39.8%)	99,616 (33.3%)	88,156 (51.1%)
Diabetes history, *n* (%)	​	​	​
No	444,853 (94.3%)	296,287 (99.1%)	148,566 (86.1%)
Yes	24,663 (5.2%)	1,628 (0.5%)	23,035 (13.3%)
Family history of cancer, *n* (%)	​	​	​
No	296,693 (62.9%)	189,981 (63.6%)	106,712 (61.8%)
Yes	164,933 (35%)	103,668 (34.7%)	61,265 (35.5%)

Abbreviation: BMl, body mass index.

## Results

### Baseline demographics

In Table 1, A total of 471,540 participants from the UK Biobank cohort with a mean age of 56.3 (SD = 8.1) years were included in the final analysis, yielding an average follow-up of 6.5 (SD = 1.2) years. Among them, 172,640 participants (36.6%) were classified as having MetS at baseline based on the JIS criteria, whereas 298,900 (63.4%) served as the non-MetS reference group. Compared with the non-MetS group, participants with MetS were slightly older (mean age: 58.5 vs. 55.1 years) and exhibited a distinct sex distribution, characterized by a higher proportion of males (53.4% vs. 41.9%). Furthermore, individuals with MetS were characterized by a more disadvantaged socioeconomic profile, including a lower proportion of college graduates (25.9% vs. 36.2%) and a higher mean IMD (19.3 vs. 16.7). As expected, those with MetS presented with pervasive metabolic derangements. The MetS group demonstrated adverse physical and metabolic derangements, including substantially higher mean body mass index (30.2 vs. 25.8 kg/m^2^), larger waist circumference (98.9 vs. 85.4 cm), elevated systolic blood pressure (144.8 vs. 136.7 mm Hg), higher triglycerides (2.3 vs. 1.4 mmol/L), and lower HDL-C (1.3 vs. 1.6 mmol/L). Regarding clinical profiles, the MetS group exhibited a concentrated prevalence of baseline diabetes (13.3% vs. 0.5%) and a greater medication burden, evidenced by increased NSAID usage (51.1% vs. 33.3%). Lifestyle-wise, participants without MetS were more likely to be never-smokers (58.4% vs. 49.6%) and reported a higher prevalence of moderate-to-high physical activity (68.6% vs. 60.3%). Missing data were transparently distributed across lifestyle and biochemical covariates, with the highest missingness observed in noncollapsible variables such as salt intake and physical activity, which were meticulously accounted for in subsequent multivariable models.

### Association between MetS, number of metabolic abnormalities, and gastric cancer risk

In the multivariable Cox proportional hazards models, baseline MetS status was positively associated with the incidence of gastric cancer, as shown in Table 2. In the fully adjusted model (model 3), participants with MetS exhibited a 36% increased risk of gastric cancer (HR = 1.36; 95% CI, 1.08–1.71) compared with those without MetS. When evaluating the cumulative burden of metabolic abnormalities, a positive trend was observed across component categories (*P* for trend = 0.033). Compared with individuals with 0 to 2 components, those with exactly three components had an increased risk (HR = 1.32; 95% CI, 1–1.70). However, the association was not strictly monotonic, and the highest-burden group (4–5 components) presented with a similar point estimate but wider CIs that crossed the null (HR = 1.31; 95% CI, 0.97–1.77), possibly due to a smaller number of cases in this extreme category.

**Table 2.** Association of MetS status and number of metabolic components with incident gastric cancer.

​Metabolic syndrome status and component count	Cases/*N*	HR (95% CI)		
		Model 1	Model 2	Model 3
MetS status	​	​	​	​
No	149/298,900	1 (Ref)	1 (Ref)	1 (Ref)
Yes	183/172,640	1.50 (1.21–1.88)***	1.37 (1.09–1.73)**	1.36 (1.08–1.71)**
MetS components^a^	​	​	​	​
0–2 components	178/325,806	1 (Ref)	1 (Ref)	1 (Ref)
3 components	91/89,619	1.44 (1.11–1.85)**	1.33 (1.03–1.72)*	1.32 (1–1.70)*
4–5 components	63/55,912	1.57 (1.18–2.10)**	1.34 (1–1.81)	1.31 (0.97–1.77)
*P* for trend	—	<0.001	0.021	0.033

NOTE: *P < 0.05; **P < 0.01; ***P < 0.001.

Model 1: Adjusted for age and sex at baseline.

Model 2: Additionally adjusted for education, ethnicity, IMD, alcohol consumption, smoking status, and NSAID use.

Model 3: Additionally adjusted for physical activity, fruit and vegetable intake, and salt intake.

a Participants with insufficient data to determine the exact number of metabolic syndrome components were excluded from the component-count analysis.

### Subsite analysis: association of metabolic syndrome with cardia and non-cardia gastric cancers

We further explored the association by anatomic subsite (Table 3). MetS was associated with a higher risk of cardia gastric cancer in the fully adjusted model (model 3, HR = 1.48; 95% CI, 1.03–2.11). For non-cardia gastric cancer, the association was directionally positive but nonsignificant after full multivariable adjustment (model 3, HR = 1.28; 95% CI, 0.95–1.73). However, formal cause-specific hazard testing did not support a statistically significant difference between cardia and non-cardia gastric cancers (*P* for heterogeneity = 0.547). These findings should be interpreted cautiously due to the limited number of subsite-specific cases.

**Table 3.** Association between MetS and gastric cancer risk by anatomic subsite.

Gastric cancer subsite and metabolic syndrome status​	Cases/*N*	HR (95% CI)		
		Model 1	Model 2	Model 3
Cardia gastric cancer	​	​	​	​
No	58/298,900	1 (Ref)	1 (Ref)	1 (Ref)
Yes	81/172,640	1.62 (1.15–2.28)**	1.49 (1.04–2.12)*	1.48 (1.03–2.11)*
Non-cardia gastric cancer	​	​	​	​
No	91/298,900	1 (Ref)	1 (Ref)	1 (Ref)
Yes	102/172,640	1.43 (1.07–1.90)*	1.30 (0.96–1.75)	1.28 (0.95–1.73)
*P* for heterogeneity	—	0.577	0.562	0.547

NOTE: *P < 0.05; **P < 0.01; ***P < 0.001.

Model 1: Adjusted for age and sex at baseline.

Model 2: Additionally adjusted for education, ethnicity, IMD, alcohol consumption, smoking status, and NSAID use.

Model 3: Additionally adjusted for physical activity, fruit and vegetable intake, and salt intake.

### Associations of individual metabolic components and dose–response patterns

To examine the associations of individual metabolic components with gastric cancer risk, we evaluated each component dichotomously (Table 4) and continuously per 1-SD increment (Table 5). Waist circumference showed the most consistent positive association; each 1-SD increase was associated with a 16% higher risk of gastric cancer (HR = 1.16; 95% CI, 1.03–1.31). The clinical threshold for central obesity was also directionally positive but did not reach statistical significance (HR = 1.21; 95% CI, 0.96–1.51). Fasting glucose, when analyzed continuously, showed a modest positive association (HR = 1.08; 95% CI, 1–1.17). In contrast, hypertension defined as a binary component was inversely associated with gastric cancer risk (Table 4). Exploratory RCS analysis suggested a possible U-shaped association between systolic blood pressure and gastric cancer risk (Supplementary Fig. S2), although this finding requires cautious interpretation and external validation.

**Table 4.** Risk of gastric cancer according to individual metabolic component categories.

​Metabolic component and status	Cases/*N*	HR (95% CI)		
		Model 1	Model 2	Model 3
Central obesity	​	​	​	​
No	194/312,591	1 (Ref)	1 (Ref)	1 (Ref)
Yes	138/157,637	1.37 (1.10–1.70)**	1.23 (0.99–1.54)	1.21 (0.96–1.51)
Hypertension	​	​	​	​
No	48/70,846	1 (Ref)	1 (Ref)	1 (Ref)
Yes	284/400,691	0.71 (0.52–0.96)*	0.70 (0.51–0.95)*	0.70 (0.51–0.96)*
Raised triglyceride	​	​	​	​
No	120/250,233	1 (Ref)	1 (Ref)	1 (Ref)
Yes	212/220,648	1.34 (1.06–1.68)*	1.24 (0.98–1.56)	1.23 (0.97–1.55)
Reduced HDL-C	​	​	​	​
No	189/325,110	Reference	1 (Ref)	1 (Ref)
Yes	143/145,557	1.25 (1–1.56)*	1.11 (0.88–1.40)	1.11 (0.88–1.39)
Hyperglycemia	​	​	​	​
No	260/400,064	1 (Ref)	1 (Ref)	1 (Ref)
Yes	72/71,476	1.22 (0.94–1.58)	1.15 (0.88–1.50)	1.14 (0.87–1.48)

NOTE: *P < 0.05; **P < 0.01.

Model 1: Adjusted for age and sex at baseline.

Model 2: Additionally adjusted for education, ethnicity, IMD, alcohol consumption, smoking status, and NSAID use.

Model 3: Additionally adjusted for physical activity, fruit and vegetable intake, and salt intake.

**Table 5.** Risk of gastric cancer per 1-SD increase in individual metabolic components.

MetS component	Cases/*N*	HR (95% CI)		
		Model 1	Model 2	Model 3
Waist circumference	332/470,228	1.25 (1.11–1.41)***	1.18 (1.04–1.33)**	1.16 (1.03–1.31)*
Systolic BP	331/470,755	0.96 (0.86–1.08)	0.96 (0.86–1.08)	0.97 (0.86–1.08)
Diastolic BP	331/470,768	1 (0.90–1.12)	1.02 (0.91–1.13)	1.02 (0.91–1.13)
Triglycerides	314/444,154	1.11 (1.01–1.23)*	1.07 (0.97–1.18)	1.06 (0.96–1.18)
HDL-C	282/407,068	0.83 (0.72–0.95)*	0.89 (0.77–1.03)	0.89 (0.77–1.04)
Fasting glucose	282/406,781	1.10 (1.02–1.19)*	1.09 (1.01–1.18)*	1.08 (1–1.17)*

NOTE: *P < 0.05; **P < 0.01; ***P < 0.001.

Abbreviation: BP, blood pressure.Model 1: Adjusted for age and sex at baseline.

Model 2: Additionally adjusted for education, ethnicity, IMD, alcohol consumption, smoking status, and NSAID use.

Model 3: Additionally adjusted for physical activity, fruit and vegetable intake, and salt intake.

### Subgroup analyses

To assess potential effect modification, we performed subgroup analyses across demographic and lifestyle strata (Supplementary Table S1). Given the modest number of incident cases (*n* = 332), stratification variables were dichotomized to preserve statistical power. The direction of the MetS–gastric cancer association was generally consistent across the examined subgroups. Formal interaction tests found no statistically significant effect modification by sex (*P* for interaction = 0.801), age (<60 vs. ≥60 years; *P* for interaction = 0.221), smoking status (*P* for interaction = 0.134), or alcohol consumption (*P* for interaction = 0.487). Thus, there was no statistical evidence that these factors modified the association between MetS and gastric cancer risk.

### Sensitivity analyses

To assess the robustness of our primary findings, we conducted several sensitivity analyses (Supplementary Table S2). A 2-year lag-time analysis yielded consistent results (HR = 1.43; 95% CI, 1.10–1.87), reducing concerns regarding potential reverse causation. The results also remained stable after further adjustment for family history of cancer and preexisting benign gastric diseases. The *H. pylori–*adjusted subcohort analysis (*n* = 8,891) was severely underpowered because only eight incident gastric cancer cases were available; therefore, no reliable inference regarding confounding by or interaction with *H. pylori* could be made.

Redefining MetS using the IDF criteria, which require central obesity as a prerequisite, yielded results consistent with the primary analysis (HR = 1.34; 95% CI, 1.07–1.68). Under the IDF framework, a significant trend was observed across categories of increasing metabolic component burden (*P* for trend = 0.017). Compared with participants with 0 to 2 components, the association did not reach statistical significance among those with exactly three components (HR = 1.16; 95% CI, 0.88–1.55), whereas participants with four to five components had an increased risk of gastric cancer (HR = 1.38; 95% CI, 1.06–1.79) in the fully adjusted model (Supplementary Table S3).

In subsite-specific analyses using the IDF definition, MetS was associated with cardia gastric cancer (HR = 1.52; 95% CI, 1.07–2.15), whereas the association with non-cardia gastric cancer was directionally positive but not statistically significant (HR = 1.22; 95% CI, 0.91–1.64; Supplementary Table S4). However, there was no statistical evidence of heterogeneity between anatomic subsites (*P* for heterogeneity = 0.375, 0.385, and 0.345 for models 1, 2, and 3, respectively). This pattern was broadly consistent with that observed in the primary analysis and supports the robustness of the overall findings under an alternative MetS definition.

## Discussion

In this large-scale prospective cohort study, we observed a modest positive association between baseline MetS and the risk of incident gastric cancer, which was generally consistent across sensitivity analyses. The risk showed a positive trend with the accumulation of metabolic components, although the association was not strictly monotonic. Subsite-specific estimates were directionally positive, with a stronger point estimate for cardia gastric cancer, but formal testing revealed no statistically significant subsite heterogeneity. Among individual components, waist circumference demonstrated the most consistent positive association, whereas exploratory RCS analyses suggested a possible nonlinear association for systolic blood pressure.

Our finding that MetS was associated with a moderately increased risk of gastric cancer is broadly consistent with several prospective studies reporting positive associations between metabolic dysfunction and gastric cancer risk (14, 18, 20). For example, the Korean Health Examinees Study reported a 26% higher risk of gastric cancer among participants with MetS and observed a positive trend with increasing numbers of MetS components (14). However, previous evidence has not been entirely consistent (12, 13, 19, 21). A meta-analysis by Mariani and colleagues (21) found no significant overall association between MetS and gastric cancer risk, although a positive association was observed among Western women. Similar to this meta-analysis, another study also found no significant association between overall MetS and gastric cancer. However, component-specific analysis revealed that reduced HDL-C was associated with an increased risk of gastric cancer specifically in males (12). Such discrepancies may be partly explained by differences in MetS definitions, population ethnicity, baseline gastric cancer risk, anatomic subtype distribution, follow-up duration, event numbers, and adjustment for lifestyle-related factors. Incomplete information on *H. pylori* infection remains an important limitation across many large-scale observational datasets, including ours. Our study adds to the existing literature by evaluating MetS in a large prospective UK cohort, exploring cardia and non-cardia gastric cancer separately with formal heterogeneity testing, and assessing individual metabolic components using both categorical and continuous approaches. These analyses suggest that the MetS–gastric cancer association is not clearly restricted to a single anatomic subsite and that central adiposity may represent one of the more consistent component-level signals, although causality and component-specific mechanisms require further confirmation.

The biological mechanisms underlying the association between MetS and gastric cancer are likely multifactorial. Central adiposity may be particularly relevant because visceral adipose tissue functions as an active endocrine and inflammatory organ rather than merely an energy-storage depot (26, 27). Excess visceral adiposity can promote chronic low-grade inflammation through altered adipokine secretion, including increased leptin and reduced adiponectin, as well as macrophage recruitment and inflammatory cytokine release (27, 28). In parallel, insulin resistance and compensatory hyperinsulinemia may influence epithelial proliferation and apoptosis through insulin and IGF-1 signaling pathways (29, 30). For proximal tumors, central obesity may additionally contribute through mechanical pathways, including increased intraabdominal pressure and gastroesophageal reflux (16, 17). Consistent with this biological framework, waist circumference showed the most consistent positive association among individual components in our analyses.

When examining anatomic subsites, our findings should be interpreted in the context of limited but growing subsite-specific evidence. Earlier studies often evaluated gastric cancer as a single endpoint or had limited capacity to distinguish cardia from non-cardia tumors. A previous UK Biobank analysis that separately examined stomach cardia and non-cardia cancer reported a more evident positive association between MetS and cardia cancer, whereas the association with non-cardia cancer was not significant (13). In contrast, evidence from East Asian cohorts, win which non-cardia tumors predominate, suggests that MetS may also be relevant to non-cardia gastric cancer, although estimation for cardia cancer is often constrained by the small number of cardia cases (14). In our analysis, MetS was associated with cardia gastric cancer, whereas the association with non-cardia gastric cancer was directionally positive but did not reach statistical significance after full adjustment. Importantly, the absence of statistically significant heterogeneity does not prove that MetS affects both subsites identically; rather, it indicates insufficient statistical evidence to conclude that the associations differ. Therefore, our findings should be viewed as broadly compatible with prior subsite-specific evidence while suggesting that a modest association with non-cardia cancer cannot be excluded. Larger pooled analyses with detailed anatomic classification and *H. pylori* information are needed to clarify whether the MetS–gastric cancer association truly differs by subsite.

Among individual metabolic components, waist circumference showed the most consistent positive association with gastric cancer risk, supporting a potential contribution of central adiposity to the observed MetS–gastric cancer association (16, 17). In contrast, most other components showed weaker or statistically imprecise associations when modeled using either clinical thresholds or continuous standardized values. Fasting glucose showed only a marginal positive association, and its interpretation should therefore remain cautious. The exploratory RCS analysis suggested a possible U-shaped association between systolic blood pressure and gastric cancer risk; however, this finding may reflect reverse causation, underlying frailty, antihypertensive treatment, comorbidity, or sparse data at the extremes of the distribution rather than a direct biological effect. Further studies with repeated biomarker measurements and longer follow-up are needed to clarify these component-specific patterns. Overall, waist circumference showed the most consistent component-level association, whereas associations for the other individual metabolic components were generally weaker or less precise.

The primary strengths of this study include its large prospective population-based design, which reduces recall bias and enables standardized assessment of metabolic biomarkers before cancer diagnosis. In addition, the study incorporated a broad range of sociodemographic, lifestyle, dietary, and medication-related covariates and used multiple complementary analytical approaches, including cumulative component burden, subsite-specific analyses with formal heterogeneity testing, alternative MetS definitions, and exploratory nonlinear modeling. These approaches allowed a more detailed characterization of the association among MetS, its components, and gastric cancer risk.

Several limitations should be acknowledged. First, *H. pylori* infection, a major determinant of gastric cancer risk, was available only in a small subcohort; with only eight incident gastric cancer cases, this analysis was severely underpowered and could not reliably assess confounding by, or interaction with, *H. pylori*. Second, because this was an observational study, residual and unmeasured confounding cannot be excluded, and causality cannot be established. Third, metabolic exposures were measured only at baseline, precluding assessment of longitudinal metabolic changes, weight change, or initiation of medications such as statins, metformin, or antihypertensive agents during follow-up. Fourth, the average follow-up of 6.5 years may be relatively short for gastric carcinogenesis and may not fully capture long-term latency. Fifth, despite the large cohort size, the number of gastric cancer cases was modest, particularly in subsite-specific and subgroup analyses, resulting in limited statistical power and wide CIs. Sixth, glucose measurements were not strictly fasting for all participants, which may have introduced exposure misclassification. Finally, UK Biobank participants are predominantly of European ancestry and are subject to healthy volunteer bias; therefore, the generalizability of our findings to populations with a higher gastric cancer burden, particularly East Asian populations, may be limited.

In this prospective UK Biobank analysis, MetS was associated with a moderately increased risk of gastric cancer. Among individual components, waist circumference showed the most consistent positive association. Subsite-specific estimates were directionally positive, with a stronger point estimate for cardia gastric cancer, although no statistically significant subsite heterogeneity was detected. These findings support further investigation of metabolic health as a potential component of gastric cancer risk assessment, whereas studies with more complete *H. pylori* data, longer follow-up, and larger numbers of subsite-specific cases are needed.

## Supplementary Material

Supplementary Figure S1 Flow diagram of the participant's enrollments

Supplementary Figure S2. Restricted cubic spline analyses of individual metabolic components and gastric cancer risk.

Supplementary Table S1. Subgroup analyses of the association between metabolic syndrome and gastric cancer risk.

Supplementary Table S2. Sensitivity analyses of the association between metabolic syndrome and gastric cancer risk.

Supplementary Table S3. Association of metabolic syndrome defined using IDF criteria and number of metabolic components with incident gastric cancer.

Supplementary Table S4. Association between metabolic syndrome defined using IDF criteria and gastric cancer risk by anatomical subsite.

## Data Availability

The present study was conducted using the UK Biobank Resource under application number 51671. The data used in this study are available to approved researchers through the UK Biobank application system. Individual-level UK Biobank data cannot be publicly shared by the authors because of participant confidentiality and UK Biobank data-access policies. The analysis code and data generated in this study are available from the corresponding author upon reasonable request, subject to UK Biobank data-access policies.
