# Supplementary material for: Metabolic Syndrome, Component Burden, and Incident Gastric Cancer Risk: A Prospective Cohort Study in the UK Biobank
Source: Cancer Res Commun. 2026 Aug 10;6(8):1889–97. doi: 10.1158/2767-9764.CRC-26-0373 (PMC13455582; doi:10.1158/2767-9764.CRC-26-0373)
Supplement: Supplementary file 1 [file crc-26-0373_supplementary_figure_s1_suppsf1.pdf]

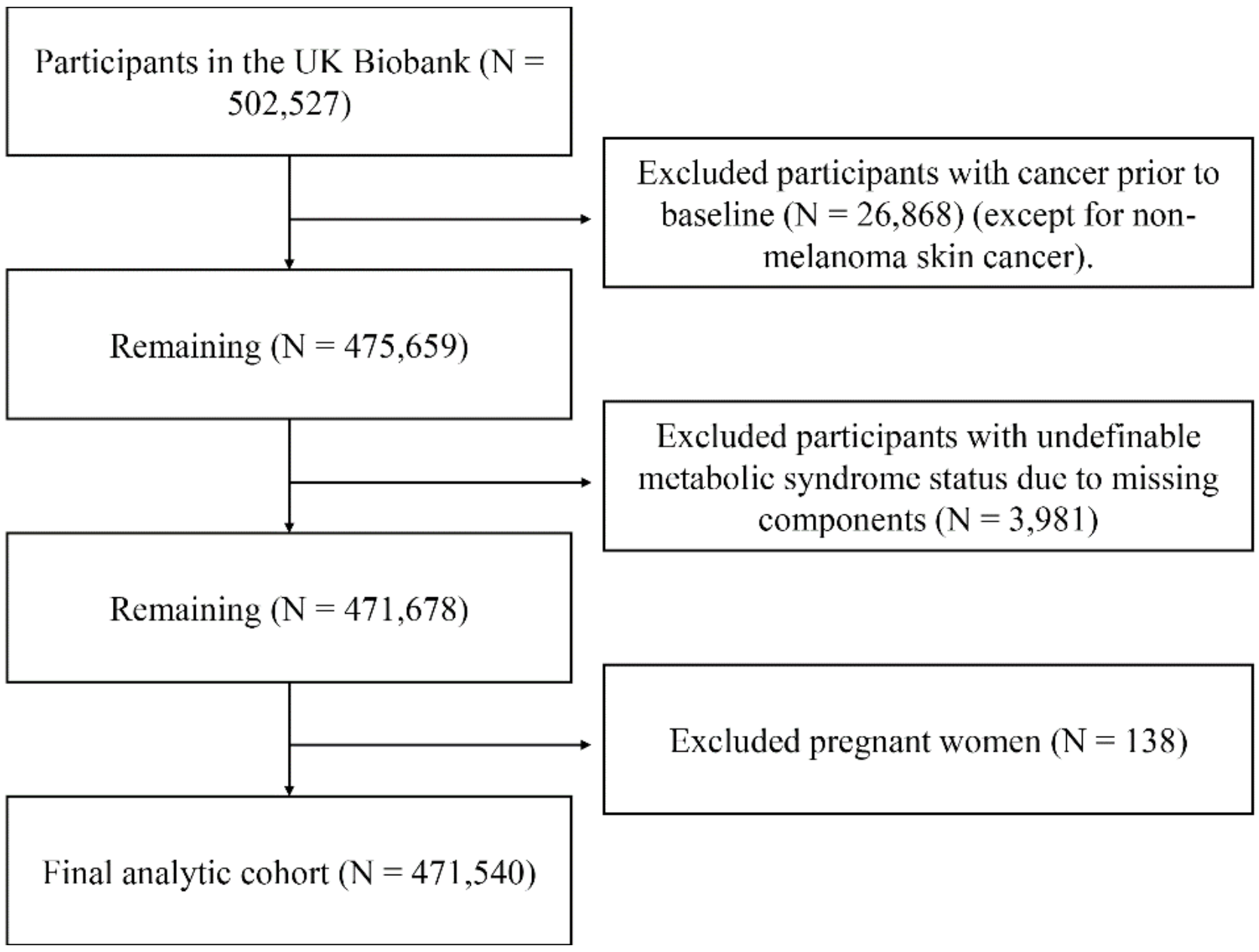

Supplementary Figure S1. Flow diagram of the participant's enrollments.
