# Supplementary material for: Metabolic Syndrome, Component Burden, and Incident Gastric Cancer Risk: A Prospective Cohort Study in the UK Biobank
Source: Cancer Res Commun. 2026 Aug 10;6(8):1889–97. doi: 10.1158/2767-9764.CRC-26-0373 (PMC13455582; doi:10.1158/2767-9764.CRC-26-0373)
Supplement: Supplementary file 2 [file crc-26-0373_supplementary_figure_s2.suppsf2.pdf]

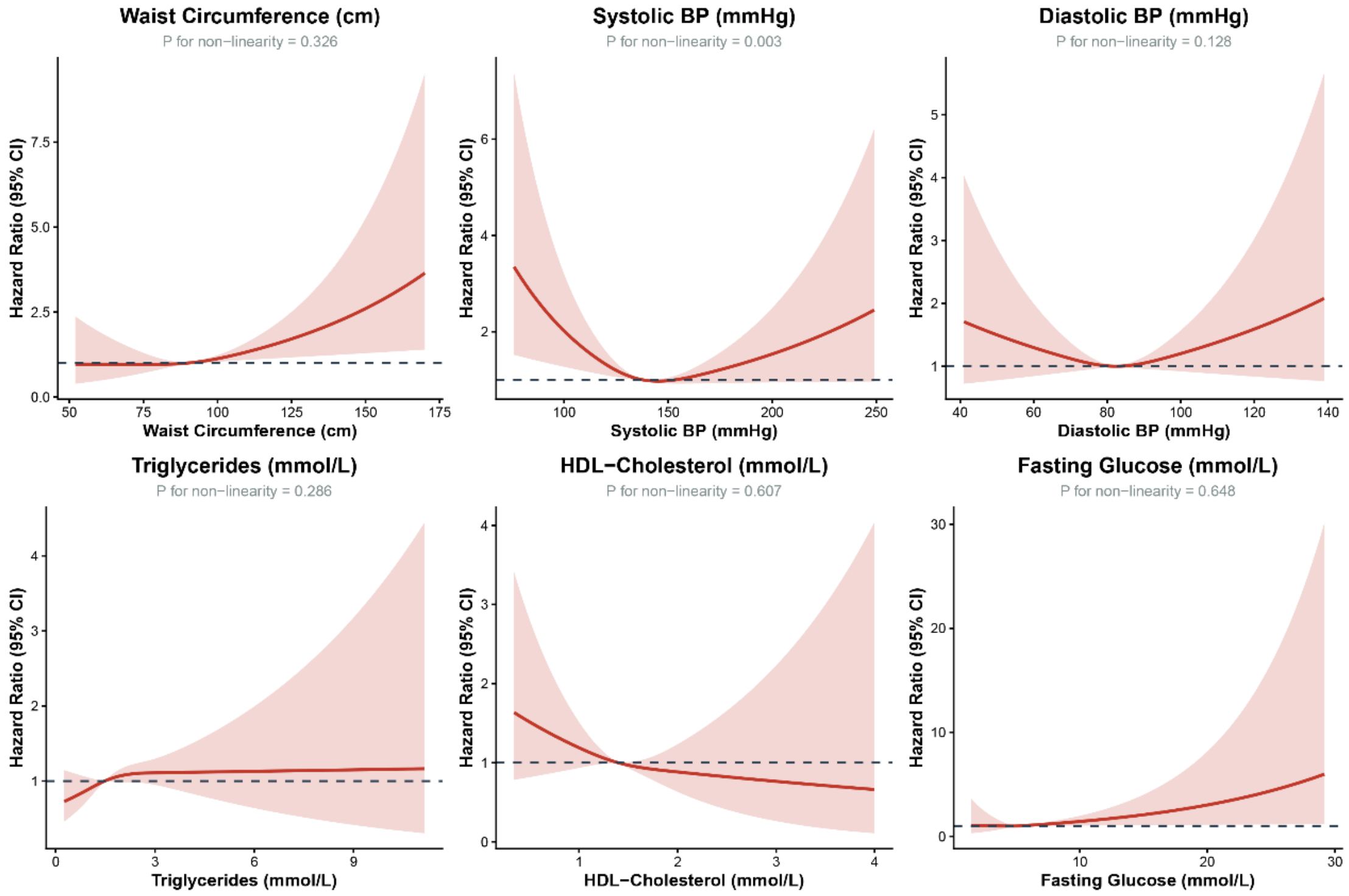

Supplementary Figure S2. Restricted cubic spline analyses of individual metabolic components and gastric cancer risk**.**

Restricted cubic spline curves showing the associations between continuous metabolic components and incident gastric cancer risk, including waist circumference, systolic blood pressure, diastolic blood pressure, triglycerides, HDL-cholesterol, and fasting glucose. Hazard ratios were estimated using Cox proportional hazards models adjusted for age, sex, ethnicity, socioeconomic deprivation, education, smoking status, alcohol consumption, NSAID use, physical activity, fruit and vegetable intake, and salt intake. Solid lines represent hazard ratios, and shaded areas represent 95% confidence intervals. Three knots were placed at prespecified percentiles of each biomarker distribution. The reference value was set at the median of each metabolic component. P values for non-linearity were calculated by comparing models with and without the non-linear spline terms.
