# Supplementary material for: Metabolic Syndrome, Component Burden, and Incident Gastric Cancer Risk: A Prospective Cohort Study in the UK Biobank
Source: Cancer Res Commun. 2026 Aug 10;6(8):1889–97. doi: 10.1158/2767-9764.CRC-26-0373 (PMC13455582; doi:10.1158/2767-9764.CRC-26-0373)
Supplement: Supplementary file 3 [file crc-26-0373_supplementary_table_s1.suppst1.docx]

**Supplementary Tables S1. Subgroup analyses of the association between metabolic syndrome and gastric cancer risk.**

| **Subgroup** | **Level** | **Cases/N  (No MetS)** | **Cases/N (MetS)** | **HR (95%CI)** | **P for interaction** |
| --- | --- | --- | --- | --- | --- |
| Sex | Female | 48/173712 | 47/80433 | 1.57 (1.03-2.40) | 0.801 |
|  | Male | 101/125188 | 136/92207 | 1.31 (1.00-1.72) |  |
| Age | <60 years | 51/192619 | 47/79618 | 1.81 (1.20-2.73) | 0.221 |
|  | >=60 years | 98/106281 | 136/93022 | 1.30 (0.99-1.71) |  |
| Smoking Status | Never/Unknown | 71/174564 | 55/85659 | 1.10 (0.76-1.60) | 0.134 |
|  | Ever smoker | 78/124336 | 128/86981 | 1.56 (1.16-2.10) |  |
| Alcohol Consumption | Non-frequent/Unknown | 86/160483 | 112/106348 | 1.33 (0.99-1.78) | 0.487 |
|  | Frequent drinker | 63/138417 | 71/66292 | 1.51 (1.06-2.17) |  |

Models were adjusted for age, sex, education, ethnicity, Index of Multiple Deprivation, alcohol consumption, smoking status, NSAID use, physical activity, fruit and vegetable intake, and salt intake, except for the corresponding stratification variable.
