# Supplementary material for: Metabolic Syndrome, Component Burden, and Incident Gastric Cancer Risk: A Prospective Cohort Study in the UK Biobank
Source: Cancer Res Commun. 2026 Aug 10;6(8):1889–97. doi: 10.1158/2767-9764.CRC-26-0373 (PMC13455582; doi:10.1158/2767-9764.CRC-26-0373)
Supplement: Supplementary file 4 [file crc-26-0373_supplementary_table_s2.suppst2.docx]

**Supplementary Table S2. Sensitivity analyses of the association between metabolic syndrome and gastric cancer risk.**

| **Analysis Models** | **Cases / N** | **HR (95% CI)^a^** |  | **P value** |
| --- | --- | --- | --- | --- |
| Primary Analysis (Model 3)^a^ | 332/471,540 | 1.36 (1.08-1.71) | | 0.008 |
| 2-year lag-time analysis^b^ | 248/471,456 | 1.43 (1.10-1.87) | | 0.008 |
| Further adjusted for family history of cancer | 332/471,540 | 1.36 (1.08-1.71) | | 0.008 |
| Further adjusted for benign gastric diseases | 332/471,540 | 1.34 (1.06-1.69) | | 0.014 |
| Sub-cohort analysis with H. pylori adjustment ^c^ | 8 / 8,891 | 2.54 (0.51-12.74) | | 0.258 |

^a^ Adjusted for age at baseline, sex, education, ethnicity, index of multiple deprivation, alcohol consumption, smoking status, NSAID use, physical activity, fruit intake, and salt intake.

^b^ Participants diagnosed within the first two years of follow-up were excluded.

^c^ Adjusted for age at baseline, sex and H. pylori due to limited cases.
