# Supplementary material for: Metabolic Syndrome, Component Burden, and Incident Gastric Cancer Risk: A Prospective Cohort Study in the UK Biobank
Source: Cancer Res Commun. 2026 Aug 10;6(8):1889–97. doi: 10.1158/2767-9764.CRC-26-0373 (PMC13455582; doi:10.1158/2767-9764.CRC-26-0373)
Supplement: Supplementary file 5 [file crc-26-0373_supplementary_table_s3.suppst3.docx]

**Supplementary Table S3. Association of metabolic syndrome defined using IDF criteria and number of metabolic components with incident gastric cancer.**

|  |  | **Hazard ratio (95% confidence interval)** | |  |
| --- | --- | --- | --- | --- |
|  | **Cases/N** | **Model 1** | **Model 2** | **Model 3** |
| MetS Status |  |  |  |  |
| No | 151/290,293 | 1.00 (Ref) | 1.00 (Ref) | 1.00 (Ref) |
| Yes | 181/180,564 | **1.48 (1.19-1.85) ***** | **1.36 (1.09-1.70) **** | **1.34 (1.07-1.68) **** |
| MetS Components |  |  |  |  |
| 0-2 components | 128/262,749 | 1.00 (Ref) | 1.00 (Ref) | 1.00 (Ref) |
| 3 components | 79/101,112 | 1.24 (0.93-1.64) | 1.17 (0.88-1.55) | 1.16 (0.88-1.55) |
| 4-5 components | 125/106,367 | **1.56 (1.21-2.01) ***** | **1.40 (1.07-1.82) *** | **1.38 (1.06-1.79) *** |
| *P* for trend | - | <0.001 | 0.013 | 0.017 |

Note: *P < 0.05; **P < 0.01; ***P < 0.001.
Abbreviations: MetS, metabolic syndrome.

Model 1: Adjusted for age and sex at baseline.

Model 2: Additionally adjusted for education, ethnicity, index of multiple deprivation, alcohol consumption, smoking status and NSAID use.

Model 3: Additionally adjusted for physical activity, fruit and vegetable intake, salt intake.

\# Participants with insufficient data to determine the exact number of MetS components were excluded from the component-count analysis.
