# Supplementary material for: Metabolic Syndrome, Component Burden, and Incident Gastric Cancer Risk: A Prospective Cohort Study in the UK Biobank
Source: Cancer Res Commun. 2026 Aug 10;6(8):1889–97. doi: 10.1158/2767-9764.CRC-26-0373 (PMC13455582; doi:10.1158/2767-9764.CRC-26-0373)
Supplement: Supplementary file 6 [file crc-26-0373_supplementary_table_s4.suppst4.docx]

**Supplementary Table S4. Association between metabolic syndrome defined using IDF criteria and gastric cancer risk by anatomical subsite.**

|  |  | **Hazard ratio (95% confidence interval)** | |  |
| --- | --- | --- | --- | --- |
|  | **Cases/N** | **Model 1** | **Model 2** | **Model 3** |
| Cardia Gastric Cancer |  |  |  |  |
| No | 58/290,293 | 1.00 (Ref) | 1.00 (Ref) | 1.00 (Ref) |
| Yes | 81/180,564 | **1.67 (1.19-2.34) **** | **1.53 (1.08-2.16) *** | **1.52 (1.07-2.15) *** |
| Non-Cardia Gastric Cancer |  |  |  |  |
| No | 93/290,293 | 1.00 (Ref) | 1.00 (Ref) | 1.00 (Ref) |
| Yes | 100/180,564 | **1.37 (1.03-1.82) *** | 1.25 (0.93-1.67) | 1.22 (0.91-1.64) |
| *P* for heterogeneity | - | 0.375 | 0.385 | 0.345 |

Note: *P < 0.05; **P < 0.01; ***P < 0.001.

Model 1: Adjusted for age and sex at baseline.

Model 2: Additionally adjusted for education, ethnicity, index of multiple deprivation, alcohol consumption, smoking status and NSAID use.

Model 3: Additionally adjusted for physical activity, fruit and vegetable intake, salt intake.
